# Truncation of *LPD1* promoter and adaptive evolution increase cytosolic acetyl-CoA supply in yeast

**DOI:** 10.1016/j.synbio.2025.10.013

**Published:** 2025-11-08

**Authors:** Ling Qin, Shoujie He, Dan Yuan, Yuyang Pan, Zhibo Yan, Mingtao Huang

**Affiliations:** School of Food Science and Engineering, South China University of Technology, Guangzhou, 510641, China

**Keywords:** *Saccharomyces cerevisiae*, *LPD1*, Acetyl-CoA, Adaptive laboratory evolution, Central carbon metabolism, Noncoding RNA

## Abstract

Acetyl-CoA is a central metabolic intermediate that serves as a key precursor for the biosynthesis of high-value compounds such as terpenoids. However, its compartmentalization within *Saccharomyces cerevisiae* limits its availability in the cytosol, constraining production of cytosol-derived metabolites. In this study, we aimed to redirect carbon flux toward cytosolic acetyl-CoA synthesis by reducing entry into the tricarboxylic acid cycle. To achieve this, we attenuated *LPD1* expression by deleting the noncoding RNA SUT526, which is located within the *LPD1* promoter region and overlaps an upstream regulatory element. This intervention impaired cell growth and hindered the utilization of non-fermentable carbon sources such as ethanol. To address this limitation, adaptive laboratory evolution was performed in ethanol-based medium, leading to rapid recovery of growth and extended cell viability. The evolved strains exhibited enhanced acetyl-CoA synthetase activity and elevated squalene production, suggesting an increased cytosolic acetyl-CoA supply. These improvements reflect enhanced flux through acetyl-CoA-dependent biosynthetic pathways. This work presents a targeted strategy for modulating central carbon metabolism to increase cytosolic acetyl-CoA supply, providing a framework for efficient production of acetyl-CoA derived compounds in yeast.

## Graphical abstract

**Figure:**
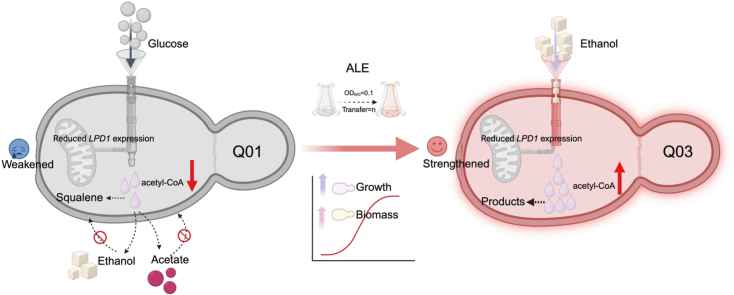

## 1 Introduction

Over the past several decades, *Saccharomyces cerevisiae* has emerged as a versatile microbial cell factory for the production of complex products, including compounds such as QS-21 that are difficult to synthesize via traditional chemical methods [1]. A key precursor in these biosynthetic processes is acetyl coenzyme A (acetyl-CoA), which not only provides essential carbon units for the synthesis of these products but also participates in the tricarboxylic acid (TCA) cycle for energy generation [2]. In yeast, acetyl-CoA metabolism is compartmentalized across the cytosol, mitochondria, and peroxisomes [3]. Despite its abundance in mitochondria, acetyl-CoA derived from this compartment is not readily accessible for cytosolic biosynthesis due to membrane impermeability and transport constraints [4]. This spatial mismatch between precursor abundance and pathway localization highlights the need for metabolic engineering strategies to increase the cytosolic acetyl-CoA supply.

Shiba et al. addressed this challenge by overexpressing *ALD6* and a mutant *Salmonella enterica* acetyl-CoA synthetase (ACS^L641P^), thereby increasing cytosolic acetyl-CoA levels and enhancing amorphadiene production [5]. Chen et al. further increased cytosolic acetyl-CoA supply by enhancing ethanol assimilation through overexpression of *ADH2*, which led to increased α-santalene production [6]. However, the compartmentalized nature of acetyl-CoA metabolism continues to pose a significant bottleneck, particularly due to the inefficiency of inter-compartmental transport [7]. Deletion of *CIT2* and *MLS1* in the glyoxylate cycle effectively reduces the loss of cytosolic acetyl-CoA pool [8]. To increase citrate supply, disruption of the *KGD1* gene, encoding α-ketoglutarate dehydrogenase in the TCA cycle, effectively redirects carbon flux toward the cytosolic acetyl-CoA through pyruvate dehydrogenase bypass [9]. This metabolic rewiring demonstrates the effectiveness of inhibiting mitochondrial respiratory metabolism to enhance precursor availability for biosynthetic applications in the cytosol.

The mitochondrial pyruvate dehydrogenase complex (PDC), which consists of three catalytic subunits (E1-E3), catalyzes the oxidative decarboxylation of pyruvate to acetyl-CoA, thereby linking glycolysis to TCA cycle [10]. Disruption of PDC subunits in *S*.*cerevisiae* has been reported to increase isobutanol production, with deletion of *LPD1* resulting in the highest titer [11]. Lpd1p also serves as a component of the glycine decarboxylase complex (GDC) [12] and 2-oxoglutarate dehydrogenase (OGDH) [13]. Due to its key role in the TCA cycle, *LPD1* deletion reduces TCA cycle flux, which may increase cytosolic pyruvate supply but often leads to impaired biomass accumulation, as indicated by reduced dry cell weight (DCW) [11,14]. Interestingly, the *LPD1* is subject to catabolite repression and is transcriptionally activated by the *HAP2*/*HAP3*/*HAP4* transcription factor complex, which relieves glucose repression under respiratory conditions [15]. However, whether modulation of the *LPD1* promoter region can redirect carbon flux away from the TCA cycle and enhance cytosolic acetyl-CoA supply remains unclear. Such redirection may improve carbon atom economy by channeling pyruvate toward cytosolic biosynthesis instead of mitochondrial oxidation.

In this study, we found that deletion of the noncoding RNA SUT526, located within the *LPD1* promoter region and overlapping a *cis*-regulatory upstream activation site (UAS), markedly reduced *LPD1* expression, leading to impaired respiratory function and reduced cell growth. However, following adaptive laboratory evolution (ALE) in ethanol-based medium, the strain recovered its growth to wild-type levels and exhibits increased cell viability. Enzymatic assays revealed increased acetyl-CoA synthetase activity in the evolved strain under ethanol conditions, promoting cytosolic acetyl-CoA synthesis. The increased cytosolic acetyl-CoA supply also contributed to increased squalene production, suggesting that metabolic rewiring following ALE can improve both growth and squalene availability for terpenoid biosynthesis.

## 2 Materials and methods

### 2.1 Strains and culture conditions

All strains used in this study are listed in Table S1, and they are derived from CEN.PK 530-1D and CEN.PK 113-5D [16]. LB medium (5 g/L yeast extract, 10 g/L tryptone, and 10 g/L NaCl) was used to culture *Escherichia coli* DH5α with or without 100 μg/mL ampicillin, at 37 °C and 250 rpm. Synthetic complete medium without uracil (SD-URA) (20 g/L glucose, 1.7 g/L yeast nitrogen base (without amino acids and (NH_4_)_2_SO_4_), 5 g/L (NH_4_)_2_SO_4_, 0.77 g/L complete supplement mixture (CSM, without uracil) was used for yeast strain selection following gene deletion. SD-FOA medium (SD-URA medium supplemented 1 g/L 5-fluoroorotic acid and 50 mg/L uracil) was used for *URA3* marker counter-selection. SD medium (SD-URA medium supplemented with 40 mg/L uracil, when required), and SE medium (10 or 20 g/L ethanol, 1.7 g/L yeast nitrogen base (without amino acids and (NH_4_)_2_SO_4_), 5 g/L (NH_4_)_2_SO_4_, 0.77 g/L complete supplement mixture) were used for adaptive laboratory evolution. YPD medium (10 g/L yeast extract, 20 g/L peptone, and 20 g/L glucose) and YPE medium (10 g/L yeast extract, 20 g/L peptone, and 20 g/L ethanol), as well as YPDT (YPD supplemented with 13.6 g/L Na_2_HPO_4_·12H_2_O, and 9.7 g/L NaH_2_PO_4_·2H_2_O to maintain pH 6.0) were used for yeast cultivation and squalene production. Solid media were prepared by adding agar to a final concentration of 2 %.

### 2.2 Plasmids, primers and genetic manipulations

The plasmids and primers used in this study are listed in Table S2 and Table S3, respectively. To construct the p426-*GPD-tHMG1* plasmid, the p426 plasmid backbone was linearized using *Eco*RI and *Hind*III, the *tHMG1* gene was amplified using primers tHMG1-OE-F/R with yeast genomic DNA as the template. the linearized plasmid backbone and gene fragment were assembled using gibson assembly, and the resulting plasmid was verified by sequencing. The deletion plasmids for *LPD1* and *SMX2* were constructed following previously described methods. The strain Q0 was used for markerless deletion of *LPD1* and *SMX2* using the GTR-CRISPR technique [17]. For construction of *ADH2* promoter deletion plasmid, primers ADH2(P)–K–F/R were used to amplify the pYZ463 plasmid using the plasmid itself as the template [18]. The PCR product was digested with the *Dpn*I, and directly transformed into yeast. The resulting plasmid was confirmed by sequencing. The pYZ463-ADH2 plasmid was then used to knock in the *TEF1* promoter at *ADH2* promoter region. Yeast transformation was performed using the LiAc/PEG method [19].

### 2.3 Transcriptome analysis

Strains Q0 and Q01 were cultivated in YPDT medium at 30 °C and 200 rpm. Cells were harvested during the exponential growth phase (OD_600_ = 1∼1.5) and washed twice with sterile water. Transcriptome sequencing, and analysis were conducted as described previously [20]. The raw sequencing data can be downloaded from the European Nucleotide Archive with access number PRJEB94411. Differential gene expression (DEG) analysis was performed using the R package DEseq2. Gene transcript levels were visualized using the Morpheus web tool (https://software.broadinstitute.org/morpheus/).

### 2.4 Metabolite quantification

For quantification of carbon metabolites (glucose, ethanol, glycerol, etc.), strains were inoculated at an initial cell density of OD_600_ ≈ 0.1 in SD medium, and cultured at 30 °C and 200 rpm under batch fermentation conditions. At designated time points, 1 mL of fermentation broth was collected and centrifuged at 10,000 rpm for 5 min to obtain the supernatant. The supernatant was filtered through a 0.22 μm membrane. Metabolite concentrations were determined using an HPLC system (Shimadzu Corporation) equipped with an Aminex HPX-87H column (Bio-Rad). The HPLC system was operated at 45 °C with a flow rate of 0.6 mL/min using 5 mM H_2_SO_4_ as the mobile phase.

### 2.5 Squalene quantification

Strains were cultivated in YPE medium at 30 °C, 200 rpm. At 96 h, 0.6 mL of fermentation broth was taken and mixed with 0.6 mL of ethyl acetate. The mixture was transferred to a tube containing 0.6 g of glass beads. Cell disruption was performed twice using a Bioprep-24R homogenizer (Allsheng) at 4 °C and 7 m/s for 1 min with an interval of 2 min. The mixture was then centrifuged at 10,000 rpm for 10 min, and the supernatant was filtered through a 0.22 μm syringe filter. Squalene quantification was performed by HPLC as described above [20].

### 2.6 Detection of cell death

Strains were cultivated in SD or SE medium at 30 °C, 200 rpm. At 96 h, 0.5 mL of fermentation broth was taken. Cells were centrifuged at 500 rpm for 5 min and the supernatant was discarded. Then, cells were were washed with cold PBS, centrifuged again under the same conditions, and then resuspended in propidium iodide (PI) solution (5 μg/mL in PBS). After incubation for 30 min at 30 °C, samples were analyzed using a flow cytometer (Beckman coulter). PI fluorescence was measured using excitation/emission wavelengths of 488/620 nm. Unstained cells were used to define the negative fluorescence threshold.

### 2.7 Transmission electron microscopy analysis

Strains were cultivated in SD medium at 30 °C, 200 rpm (initial OD_600_ ≈ 0.1). At 24 h, cells were harvested and fixed in 2.5 % glutaraldehyde at 4 °C overnight. The fixed samples were washed with PBS twice. Centrifuged and discarded the supernatant, dehydrated with a graded ethanol series. Replace with isovaleryl acetate for over 3 h. Dry the samples using critical point drying with carbon dioxide, sputter coat with gold, and then observe and photograph under a transmission electron microscope (JEOL, JEM-1400 Flash).

### 2.8 Adaptive laboratory evolution

The ALE process for Q01 strain is illustrated in Fig. 3b. In the first phase, four independent Q01 colonies were cultivated in SD medium at 30 °C and 220 rpm for 24 h. The cultures were then transferred into fresh SD medium with an initial OD_600_ of 0.1 and propagated for 12 successive cycles. In the second phase, the strains were transferred to the SE medium containing 10 g/L ethanol with an initial OD_600_ of 0.1 or 0.05, and propagated for 14 additional cycles. Every two transfers, the strains were preserved and verified by PCR. The evolution process continued until the strains were able to grow normally in a SE medium containing 10 g/L ethanol as the sole carbon source (reaching an OD_600_ of 8–10 within 96 h).

The ALE process for strain Q07 is illustrated in Fig. S5a. Four independent colonies were cultivated in SE medium containing 10 g/L ethanol at 30 °C, 220 rpm for 24 h. The cultures were transferred into the same medium with an initial OD_600_ of 0.1 and propagated for 51 cycles. Evolution was terminated on day 51 due to the absence of detectable growth.

### 2.9 ATP and acetyl-CoA synthetase activity assays

Strains Q06 and Q12 were cultivated in YPE medium at 30 °C, 200 rpm. Cells were collected from culture when the cell density reached OD_600_ ≈ 3–4. Then, the cell pellets were used to measure ATP levels and acetyl-coenzyme A synthetase activity using the BacTiter-Glo™ Microbial Cell Viability Assay (Cat No. G8230, Promega, USA) and the Amplex Red Acetyl-Coenzyme A Synthetase Assay Kit (Cat No. S0391S, Beyotime, China), according to the manufacturer's recommendations. The acetyl-CoA content was determined using the Acetyl-CoA Content Assay Kit (Cat No. BC0908, Beyotime, China ) according to the manufacturer's recommendations.

### 2.10 Whole genome sequencing and analysis

Whole-genome sequencing was performed on both the evolved strains and the parental control strain Q0. Strains were cultured overnight in 50 mL of YPD medium to an OD_600_ ≈ 3–4. Genomic DNA was extracted using the Rapid Fungi Genomic DNA Isolation Kit (NO. B518229, Sangon Biotech, China). Sequencing and downstream bioinformatic analyses were carried out by Sangon Biotech (Shanghai, China). The raw sequencing data can be downloaded from the European Nucleotide Archive with access number PRJEB101288. Sequence reads were aligned to the reference genome of *S. cerevisiae* CEN.PK 113-7D, and all identified mutations in the evolved strains are summarized in Table S4.

### 2.11 Statistical analysis

Experiments were performed with at least three biological replicates. Statistical analysis was conducted using two-tailed *t*-test and analysis of variance in Microsoft Excel 2019. All the data are presented as mean ± SD, * marked as *P* < 0.05, ** marked as *P* < 0.01 and *** marked as *P* < 0.001.

## 3 Results

### 3.1 Loss of SUT526 noncoding sequence reduces cell density

**Fig. 1:**
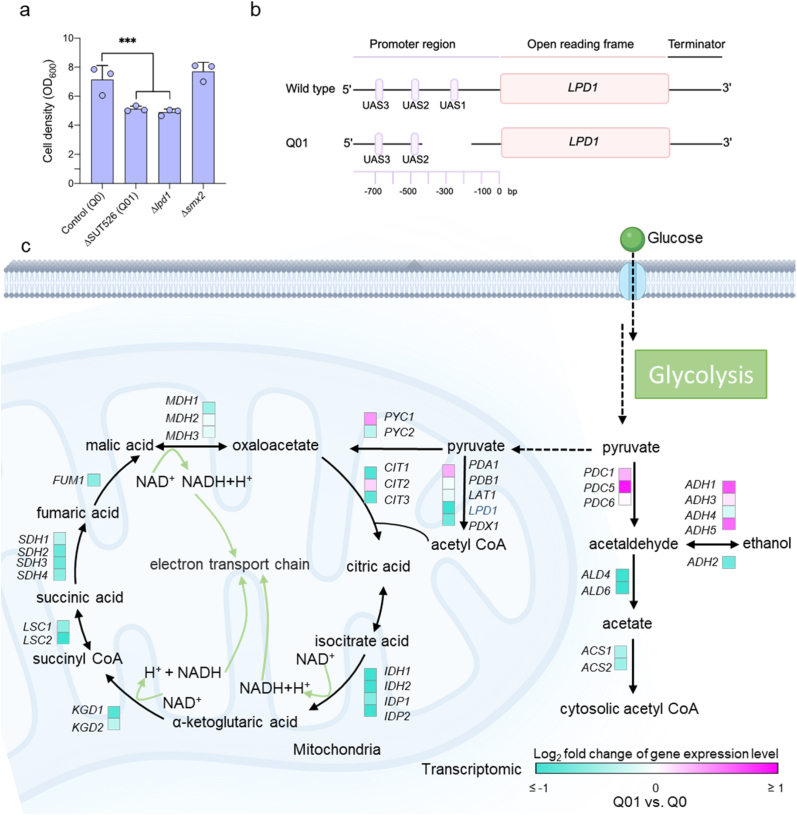
Low *LPD1* expression limits carbon flux through the TCA cycle. **a**. Cell density of control and mutant strains cultured in SD medium at 30 °C for 96 h. **b**. Schematic representation of the *LPD1* promoter region in wild-type and Q01 strains. **c**. Transcriptional changes of genes involved in central carbon metabolism. Data are presented as mean ± SD (n = 3 biologically independent samples). The statistical significance was determined by a two-tailed homoscedastic (equal variance) *t*-test. ***P < 0.001.

Genome-wide screening for intergenic long noncoding RNAs (lincRNAs) previously identified that deletion of SUT526 results in growth defects [20]. SUT526 is located within the promoter region of *LPD1* and *SMX2* (Fig. S1a). To assess whether the loss of SUT526 influences the expression of these adjacent genes, individual knockouts of *LPD1* and *SMX2* were constructed. Growth defects were observed only in the *LPD1* knockout strain, indicating that the phenotype is primarily associated with *LPD1* loss (Fig. 1a and S1b). Notably, deletion of SUT526 in the Q01 strain also removes a *cis*-regulatory element known as upstream activation sites (UAS) (Fig. 1b). This UAS serves as a binding site for the HAP2/HAP3/HAP4 transcription factor complex, which mediates derepression of *LPD1* (Fig. S1c), allowing its transcription once glucose repression is lifted [15]. We independently deleted the three UAS elements in the *LPD1* promoter and found that their removal reduced *LPD1* expression levels (Fig. S1d–f). However, the effect on growth differed from that observed in Q01.

To determine the effect of SUT526 deletion on *LPD1* transcription, RNA-seq analysis was performed on the Q01 strain (Fig. S2a). The results revealed that the expression level of *LPD1* in the Q01 strain during exponential growth reduced approximately to one-eighth of that in the control strain (Fig. S2b). Reduced *LPD1* expression limits the mitochondrial conversion of pyruvate to acetyl-CoA, thereby restricting precursor supply for TCA cycle. This constraint was accompanied by a broad downregulation of TCA cycle-associated genes (Fig. 1c). Transcriptomic profiling of the Q01 strain further showed that over 1600 genes exhibited more than a two-fold reduction in expression (Fig. S2c), suggesting a systemic impact likely linked to impaired TCA cycle function and reduced energy production. These findings indicate that the absence of SUT526 disrupts *LPD1* transcription, constrains mitochondrial acetyl-CoA biosynthesis, impairs energy metabolism, and ultimately leads to growth defects.

### 3.2 Q01 displays impaired utilization of non-fermentable carbon sources

**Fig. 2:**
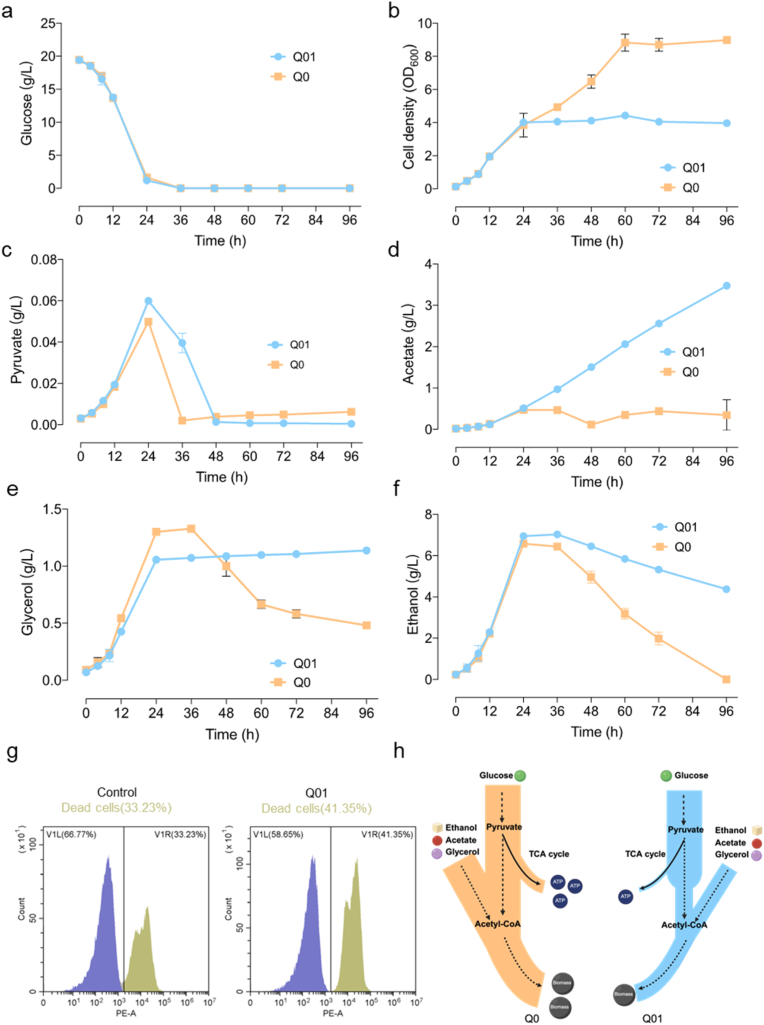
Physiological characterization of the Q01 strain. **a**. Glucose consumption. **b**. Cell density. **c**. Pyruvate production. **d**. Acetate production. **e**. Glycerol production. **f**. Ethanol production. Strains were cultivated in SD medium at 30 °C for 96 h. **g**. Representative flow cytometry plots showing the proportion of dead cells in strains. **h**. Impaired utilization of non-fermentable carbon sources in Q01 limits biomass accumulation.

The attenuation of the TCA cycle in the Q01 strain is expected to alter intracellular carbon flux distribution. To assess the strain's carbon source utilization, batch fermentations were conducted. Both Q01 and the control strain fully consumed glucose within 36 h (Fig. 2a). However, following glucose depletion, the growth of the Q01 strain plateaued, while the control strain continued to grow, ultimately reaching a cell density approximately twice as high as that of the Q01 strain (Fig. 2b). During the glucose phase, Q01 exhibited slightly increased pyruvate production compared to the control, consistent with downregulated expression of TCA cycle genes and suggesting impaired mitochondrial carbon oxidation (Fig. 2c). In the Q01 strain, both acetate and glycerol accumulated without subsequent consumption, accompanied by significantly reduced expression of the *ALD4* and *ALD6*. This likely contributes to limited cytosolic acetyl-CoA availability, thereby hindering biomass formation (Fig. 1, Fig. 2d, e). By 96 h, the Q01 strain had only partially consumed ethanol, leaving a residual concentration of 4.3 g/L, significantly higher than that in the control (Fig. 2f). Additionally, cell viability declined, with Q01 showing a dead cell proportion of 41.35 %, compared to 33.23 % in the control strain (Fig. 2g). The Q01 strain's inability to effectively utilize non-fermentable carbon sources thus results in a significant shortage of precursors required for biomass biosynthesis (Fig. 2h). These findings indicate that reduced capacity of the Q01 strain to utilize non-fermentable carbon sources is a key contributor to its growth impairment.

**Fig. 3:**
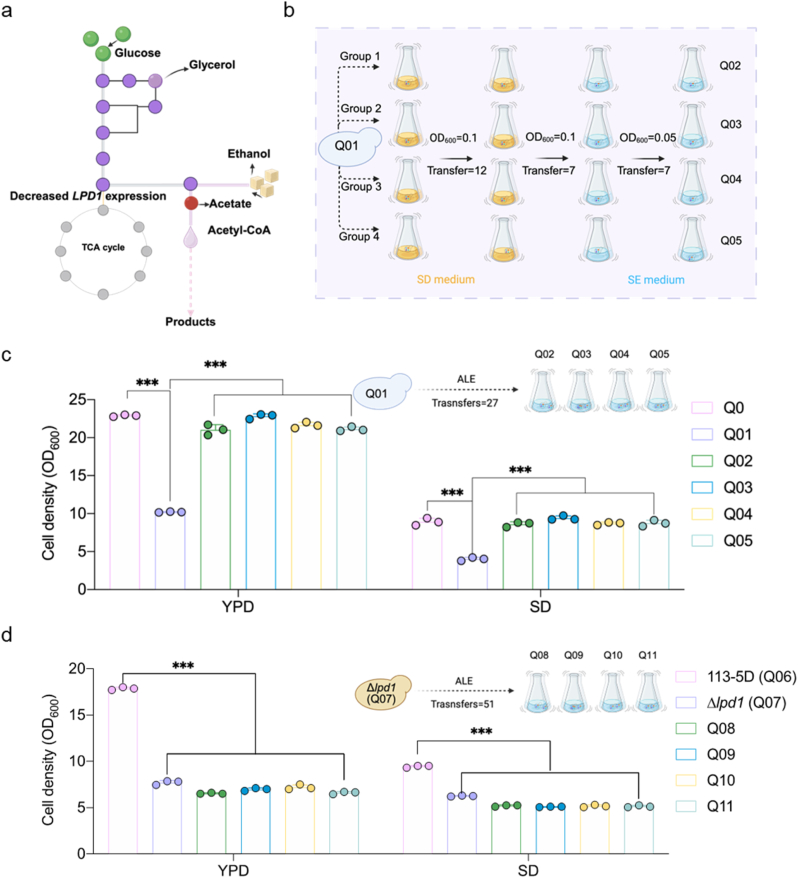
Adaptive laboratory evolution (ALE) enabled restoration of cell growth. **a**. Strategy for stepwise carbon source feeding in ALE, transitioning from glucose to ethanol to enhance cytosolic acetyl-CoA supply under reduced *LPD1* expression. **b**. Schematic overview of the ALE procedure. Four independent Q01 colonies were first cultured in SD medium (20 g/L glucose) and then transferred into SE medium (10 g/L ethanol) under varying initial OD_600_ conditions. Serial transfers yielded evolved strains Q02-Q05. **c**. Cell density of control, Q01 and evaluation strains in SD and YPD media at 30 °C for 96 h. **d**. Cell density of control, *Δlpd1* and evaluation strains in SD and YPD media at 30 °C for 96 h. Data are presented as mean ± SD (n = 3 biologically independent samples). The statistical significance was determined by a two-tailed homoscedastic (equal variance) *t*-test. ***P < 0.001.

### 3.3 Restoration of cell growth by adaptive laboratory evolution

Given that the Q01 exhibits an impaired ability to utilize non-fermentable carbon sources, potentially limiting cytosolic acetyl-CoA synthesis, we hypothesized that enhancing cytosolic acetyl-CoA production could restore cell growth. To test this, we applied ALE to improve the strain's ability to utilize non-fermentative carbon sources (Fig. 3a).

In the first stage, four independent Q01 colonies were cultured in SD medium containing 20 g/L glucose (Fig. 3b). We reasoned that initiating evolution under moderate selective pressure would facilitate early adaptation, and that glucose, by promoting cytosolic acetyl-CoA production via glycolysis, could help establish a more favorable metabolic state for subsequent adaptation. However, after 12 serial transfers, the evolved strains exhibited only partial restoration of growth (Fig. S3a and b). We hypothesized that direct selection for non-fermentable carbon source utilization might accelerate growth recovery, as fewer metabolic steps are required to generate cytosolic acetyl-CoA from ethanol compared with glucose (Fig. 3a). In the second stage, the strains were transferred to SE medium containing 10 g/L ethanol. After five transfers, significant growth improvement was observed (Fig. S3a–c). On day 27, evolution was terminated, and all four evolved lineages (Q02-Q05) achieved growth comparable to the control strain (Fig. 3c).

Sequencing confirmed that the SUT526 deletion remained intact in the evolved strains, prompting whole-genome sequencing to identify potential compensatory mutations (Fig. S4a). Whole-genome sequencing of one high-performing mutant from each of the four evolved populations revealed mutations in the evolved clones (see Table S4 for details). These mutations were validated by PCR amplification and sequencing (Fig. S4b). *SEC27*, which encodes a component of the COPI complex involved in retrograde transport between the ER and Golgi apparatus [21], was mutated in all four strains (SEC27^D677Q^). Similarly, *NUP133*, a gene required for nuclear protein import and tRNA export [22], was mutated at position G685D (NUP133^G685D^) in all evolved strains. In addition, a mutation in *CDC24* (E239Q), a gene essential for cell polarity establishment and maintenance [23], was identified in strains Q02 and Q05. We hypothesized that these mutations contribute to the restored growth phenotype. However, reverse metabolic engineering of the identified individual mutations, as well as their combinations, failed to recover growth in the Q01 background (Fig. S4c). Among the evolved strains, Q03 had the fewest identified mutations and was selected for further analysis due to its simpler mutation profile (Fig. S4b). To evaluate phenotypic stability, Q03 was passaged for seven additional transfers (14 days) in glucose-based SD medium. Its growth remained stable and comparable to that of Q03 prior to passaging, confirming phenotypic stability (Fig. S4d). It is possible that relevant mutations located in repetitive or poorly mapped regions were not detected by sequencing, or that post-translational modifications affecting regulatory proteins also contribute to the restored growth phenotype.

Having observed that the strain Q01with low *LPD1* expression could regain growth in SE medium through ALE, we next asked whether a complete *LPD1* deletion strain (Q07) retained a similar capacity for adaptation (Fig. S5a). However, after an extended evolution period of 51 days, the resulting strains (Q08, Q09, Q10 and Q11) failed to recover growth, suggesting that a basal level of TCA cycle activity is essential for successful evolutionary adaptation (Fig. 3d and S5b).

These results indicate that ALE in ethanol-based medium can efficiently restore growth in strains with severely reduced *LPD1* expression, but complete loss of TCA cycle function impairs adaptive recovery under these conditions.

### 3.4 Physiological characterization of the evolved strain Q03

**Fig. 4:**
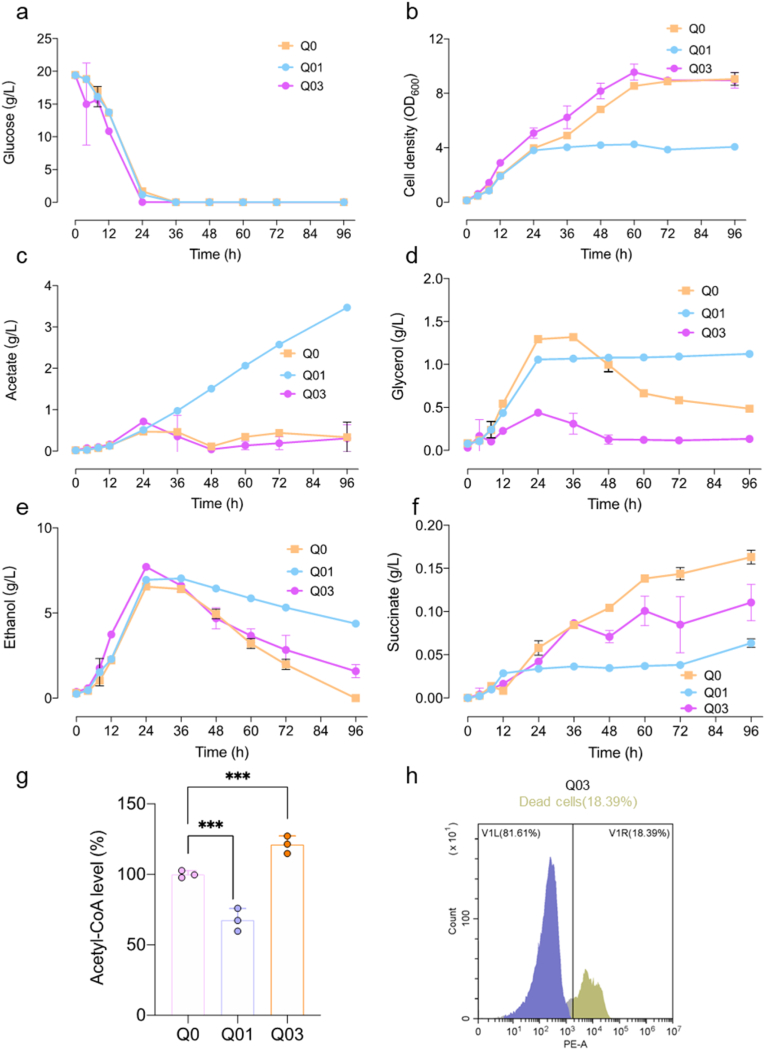
Characterization of physiological traits in evolved strains. **a**. Glucose consumption. **b**. Cell density. **c**. Acetate production. **d**. Glycerol production. **e**. Ethanol production. **f**. Succinate production. Strains were cultivated in SD medium at 30 °C for 96 h. **g**. Acetyl-CoA concentration is shown relative to Q0, which was set as 100 %. **h**. Representative flow cytometry plot showing the proportion of dead cells in Q03. Data are presented as mean ± SD (n = 3).

To investigate the physiological and metabolic adaptations of the evolved strains, batch fermentations were conducted using the evolved strain Q03, along with the parental strain Q0 and the unevolved mutant Q01. The evolved strain Q03 exhibited faster consumption of glucose, glycerol, and acetate compared with the control strain, resulting in more rapid early growth. Ultimately, both strains reached similar final cell densities, indicating that Q03 effectively utilizes non-fermentable carbon sources to support growth (Fig. 4a–d). Additionally, Q03 showed significantly improved ethanol utilization compared to Q01, reflecting a more balanced redistribution of intracellular carbon flux. Although succinate production in Q3 was elevated compared to Q01, it remained lower than in Q0, consistent with limited TCA cycle activity due to reduced *LPD1* expression (Fig. 4e and f). This constraint likely redirects carbon flux towards cytosolic acetyl-CoA synthesis. As shown in Fig. 4g, the acetyl-CoA concentration in Q03 was significantly higher than in Q0, supporting this hypothesis. Q03 maintained normal cellular morphology during fermentation (Fig. S6a). After 96 h of fermentation, Q03 showed significantly lower cell death rates than Q0, indicating enhanced physiological robustness (Fig. 4h). Furthermore, when cultivated in medium containing 20 g/L ethanol as the sole carbon source, the Q03 strain showed a lower mortality rate than the control strain (Fig. S6b), further supporting its improved stress tolerance under non-fermentative growth conditions. These results suggest that Q03 has undergone beneficial adaptations enabling efficient utilization of non-fermentable carbon sources and improved physiological stability, supporting its potential as a robust microbial cell factory.

### 3.5 Evolved strains enhance cytosolic acetyl-CoA supply and improve squalene production

**Fig. 5:**
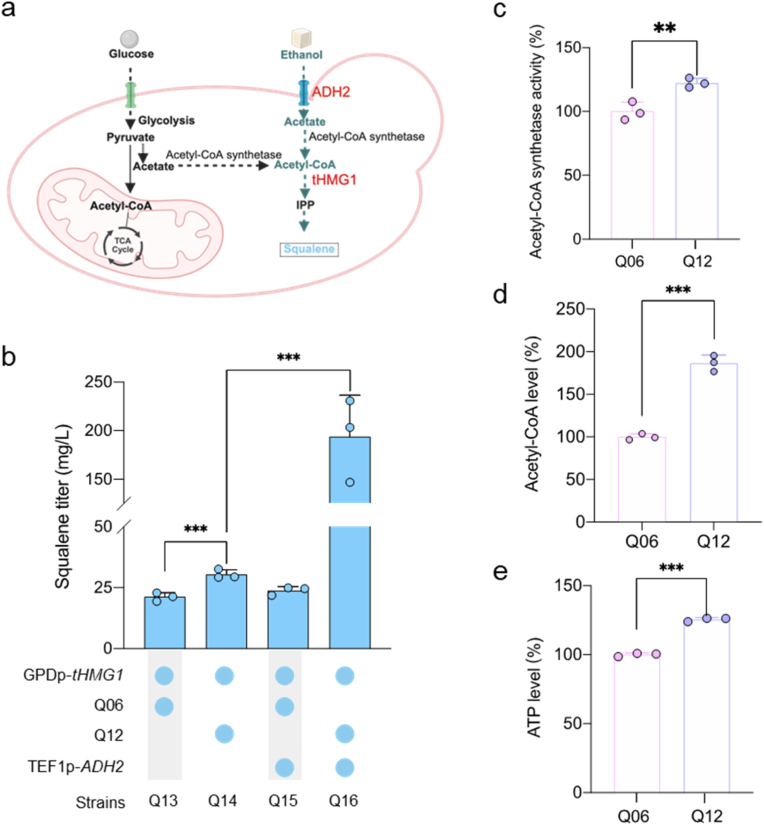
Evaluation of evolved strains for enhanced squalene production. **a**. Schematic diagram of squalene synthesis, red indicates overexpressed genes. **b**. Squalene titers of engineered strains, strains were cultivated in YPE media at 30 °C for 96 h for squalene production. **c**. Acetyl CoA synthetase activity is shown relative to Q06, which was set as 100 %. **d**. Acetyl CoA level is shown relative to Q06, which was set as 100 %. **e**. Intracellular ATP level is shown relative to Q06, which was set as 100 %. strains were cultivated in YPE media at 30 °C for 24 h (OD_600_ ≈ 3–4. Data are presented as mean ± SD (n = 3). The statistical significance was determined by a two-tailed homoscedastic (equal variance) t-test. **P < 0.01; ***P < 0.001.

Strain Q03 lacks *TPI1* (encoding for triose phosphate isomerase), which enables stable maintenance of recombinant expression plasmids using the *Schizosaccharomyces pombe POT1* gene (a heterologous *TPI1* gene) as a selection marker [24]. To broaden the applicability of the evolved strain as a chassis for producing diverse compounds, *TPI1* gene was reintroduced at its native locus to allow for plasmid curing. This modification generated strain Q12, in which the pCP-Aamylase plasmid was successfully eliminated. As presented in Fig. S7, the *LPD1* gene expression was still significantly lower in strain Q12 relative to strain Q06. In *S. cerevisiae*, squalene is a key precursor for many terpenoids and is synthesized from acetyl-CoA via the endogenous mevalonate pathway [4]. To evaluate whether cytosolic acetyl-CoA availability was improved in the evolved strain, a plasmid overexpressing a truncated hydroxymethylglutaryl-CoA synthase (*tHMG1*) was introduced into both the reference (Q06) and the evolved (Q12) strains (Fig. 5a and S8). The resulting strain Q14 (derived from Q12) exhibited significantly higher squalene production than Q13 (derived from Q06) (Fig. 5b). We speculate that the increased cytosolic acetyl-CoA supply contributed to the enhanced squalene production. This is supported by enzymatic assays showing higher acetyl-CoA synthetase activity in Q12 compared with Q06 (Fig. 5c). Furthermore, Q12 showed a significantly higher intracellular acetyl-CoA level than Q06 (Fig. 5d). To further enhance ethanol utilization and acetyl-CoA supply, the *ADH2* promoter was replaced with the *TEF1* promoter. This modification led to a significant increase in squalene production in strain Q16, reaching 193.7 mg/L, whereas strain Q15 showed no significant improvement (Fig. 5a and b). The enhanced performance of Q16 may be attributed to the increased ATP synthesis capacity observed in Q12 (Fig. 5e), which likely facilitates the ATP-dependent condensation of acetate and coenzyme A, further increasing cytosolic acetyl-CoA levels. These results indicate that evolved strains exhibit enhanced acetyl-CoA synthetase activity and ATP synthesis capacity, which together improve cytosolic acetyl-CoA supply and supporting increased production of acetyl-CoA derived metabolites such as squalene.

## 4 Discussion

Redirecting metabolic flux towards cytosolic acetyl-CoA by engineering the central carbon metabolism has become a key strategy in yeast metabolic engineering [25]. Strains with increased acetyl-CoA availability serve as versatile platforms for the heterologous production of acetyl-CoA derived compounds, including terpenoids used in pharmaceuticals, biofuels, flavoring agents [26,27]. One previously reported approach involves disrupting *KGD1* to redirect carbon flux from the pyruvate dehydrogenase bypass toward acetyl-CoA synthesis [9]. However, this intervention often leads to excessive accumulation of acetate, which disrupts cellular metabolism and reduces efficiency. Given that the PDH bypass also results in CO_2_ release and carbon atom loss, we instead pursued attenuation of *LPD1* expression to restrict carbon flux into the TCA cycle (Fig. 1). Nonetheless, we observed continuous acetate accumulation (Fig. 2d), which is known to exert cytotoxic effects on yeast cells [28]. Specifically, in Q01, ethanol is only partially consumed, with a substantial proportion of the utilized ethanol converted to acetate, which subsequently accumulates due to inefficient assimilation. As a result, the carbon derived from ethanol is predominantly redirected to acetate accumulation rather than being effectively incorporated into central metabolic pathways. This metabolic bottleneck limits the supply of acetyl-CoA and energy, ultimately restricting both biomass formation and product synthesis (Fig. 2f and h). ALE is a powerful strategy for accelerating strain adaptation under adverse environmental conditions [29]. This method has been successfully applied to enhance the utilization of unconventional carbon sources, such as methanol, enabling high production of fatty acids and other metabolites [30,31]. In this study, the Q01 strain with reduced *LPD1* expression quickly regained growth capability after ALE (Fig. 3c). In contrast, the Q07 strain, in which the entire *LPD1* open reading frame was deleted, failed to exhibit any growth recovery even after 51 days of evolution. These results suggest that a minimal level of *LPD1* expression is necessary for successful adaptation. This is likely due to the indispensable role of certain TCA cycle intermediates in amino acid biosynthesis [32]. Compared with Q01, the evolved strain Q03 showed increased succinate production between 24 and 96 h, which may reflect improved utilization of non-fermentable carbon sources. We speculated that enhanced cytosolic acetyl-CoA synthesis in Q03 could partially support mitochondrial metabolism by increasing the supply of TCA cycle substrates, potentially via citrate or other intermediates. However, this observation alone does not provide direct evidence of increased TCA cycle activity.

Metabolic rewiring in the evolved strains also resulted in extended cell lifespans across different carbon sources. Increased cytosolic acetyl-CoA levels were confirmed by both squalene production and elevated acetyl-CoA synthetase activity (Fig. 5). As a representative acetyl-CoA-derived metabolite, squalene not only reflects improved precursor availability but also serves as a platform molecule for synthesizing high-value terpenoids such as retinol [33,34], carotenoids [35] and ursolic acid [36].

In summary, we developed a metabolic engineering strategy that attenuates TCA cycle flux via *LPD1* promoter truncation and restores growth through adaptive evolution. This approach enhances cytosolic acetyl-CoA availability without fully disrupting respiratory function, thereby improving carbon atom economy. The evolved strains exhibited increased acetyl-CoA synthetase activity and higher squalene titers, indicating enhanced acetyl-CoA supply and metabolic flexibility. These findings establish an effective framework for redirecting central carbon flux to support efficient biosynthesis of acetyl-CoA derived compounds, particularly those relying on cytosolic pathways.

## CRediT authorship contribution statement

**Ling Qin:** Conceptualization, Formal analysis, Investigation, Methodology, Writing – original draft. **Shoujie He:** Conceptualization, Formal analysis, Investigation, Validation, Writing – original draft. **Dan Yuan:** Investigation. **Yuyang Pan:** Investigation. **Zhibo Yan:** Formal analysis. **Mingtao Huang:** Conceptualization, Formal analysis, Project administration, Supervision, Writing – review & editing, Funding acquisition.

## Declaration of competing interest

The authors declare that they have no known competing financial interests or personal relationships that could have appeared to influence the work reported in this paper.
