# Supplementary material for: Truncation of LPD1 promoter and adaptive evolution increase cytosolic acetyl-CoA supply in yeast
Source: Synth Syst Biotechnol. 2025 Nov 8;12:10–9. doi: 10.1016/j.synbio.2025.10.013 (PMC12639635; doi:10.1016/j.synbio.2025.10.013)
Supplement: Multimedia component 1 [file mmc1.pdf]

Supplementary information for

Truncation of *LPD1* promoter and adaptive evolution increase cytosolic acetyl-CoA supply in yeast

Ling Qin[a,1], Shoujie He[a,1], Dan Yuan[a], Yuyang Pan[a], Zhibo Yan[a], and Mingtao Huang[a,*]

[a]School of Food Science and Engineering, South China University of Technology, Guangzhou, 510641, China

[1]These authors contributed equally to this work.

[*] To whom correspondence should be addressed. Email: huangmt@scut.edu.cn.

This file includes:

Fig. S1 to Fig. S8
Table S1 to Table S3

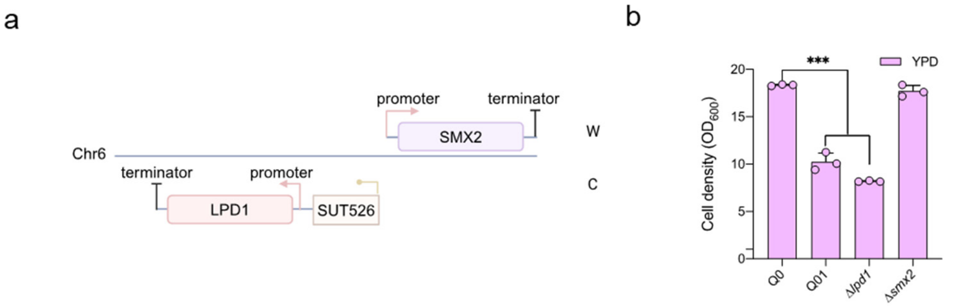

c

HAP2/HAP3/HAP4 binding elements

Match to consensus 5'-TNA/GTTGGT-3'	Gene	Gene product	Distance from ATG start codon (bp)
TCATTGGc			-204
TCATTGGc	*LPD1*	Lipoamide dehydrogenase	-497
TTATTGGc			-731

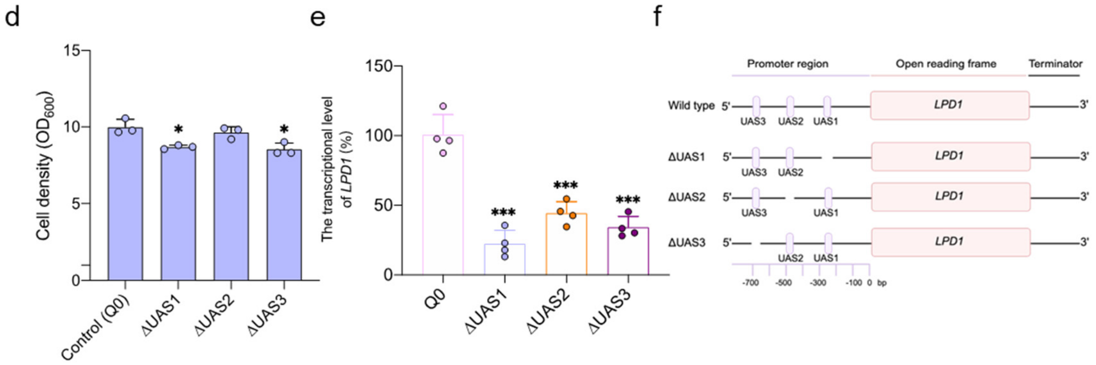

Fig. S1. Investigation of SUT526 and regulatory elements in the *LPD1* promoter region.

a. Genomic context of SUT526, located between *LPD1* and *SMX2*. **b**. Cell density of strains in YPD medium at 30 °C for 96 h. **c**. HAP2/HAP3/HAP4 binding elements (adapted from *Bowman* et al, *Mol Gen Genet.* 1992;231(2):296-303.). **d**. Cell density of strains in SD medium at 30 °C for 96 h. **e**. The transcriptional level of *LPD1*, with the starting strain Q0 as the control. **f**. Schematic representation of the *LPD1* promoter region in wild-type, ΔUAS1, ΔUAS2 and ΔUAS3 strains. The statistical significance was determined by a two-tailed homoscedastic (equal variance) *t* test. * $P < 0.05$; *** $P < 0.001$.

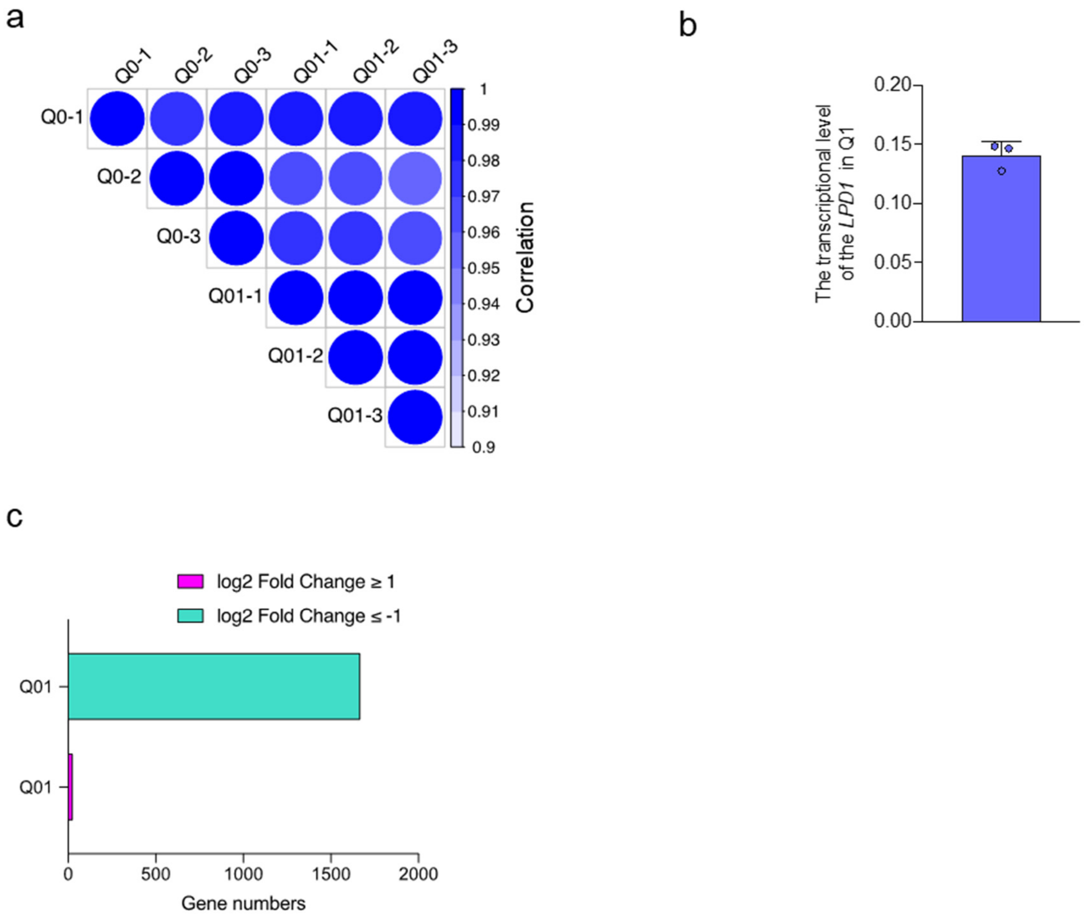

Fig. S2. Transcriptome analysis of strains Q0 and Q01.

a. Pearson correlation matrix of transcriptomic profiles from biological replicates of control (Q0) and engineered strain Q01. **b**. The transcriptional level of *LPD1* in Q01, with the starting strain Q0 as the control. **c**. Differentially expressed genes (significantly DEGs, P-adj <0.05 and $|\log_2$ Fold change$| \geq 1$) in strain Q01 compared with the control Q0.

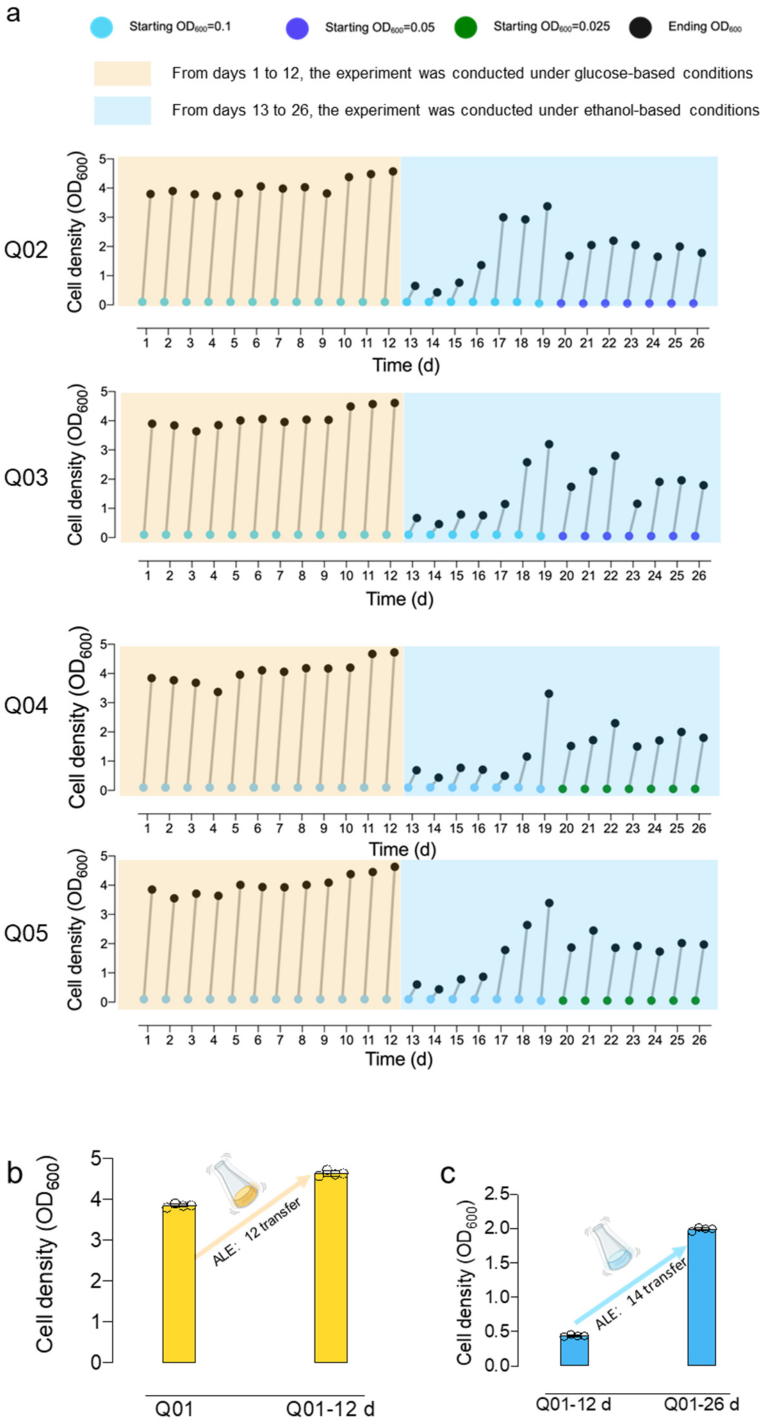

Fig. S3. Adaptive laboratory evolution (ALE) facilitates growth recovery of strain Q01.

a. Serial passaging during ALE. Cultures were transferred daily when the OD_{600} reaches 3-4 within 24 h in SD medium (20 g/L glucose). After 12 days of passaging, cells were switched to SE medium (10 g/L ethanol) and further evolved until growth was restored. Color-coded points represent the starting OD_{600} (0.1 or 0.05) and the corresponding final OD_{600} of each cycle. **b**. Cell density of the Q01 strain before and after 12 days of ALE in SD medium, measured after 24 h of cultivation at 30 °C. c. Cell density of evolved strains from day 12 and day 26 of ALE, measured in SE medium (10g/L ethanol) at 30 °C for 24 h. Data are presented as mean ± SD (n=4).

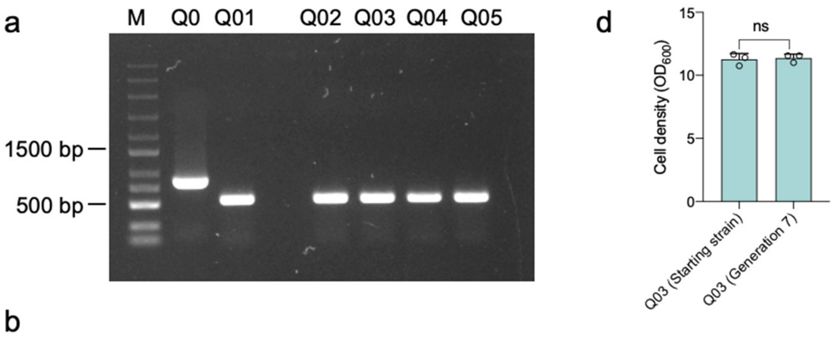

b

Missense	Gene	*SEC27*	*NUP133*	*CDC24*	*DRS2*
	**Amino acid mutations**	D677Q	G685D	E239Q	K944T
**Evolution strains**	**Q02**	M	M	M	
	**Q03**	M	M		
	**Q04**	M	M		M
	**Q05**	M	M	M	

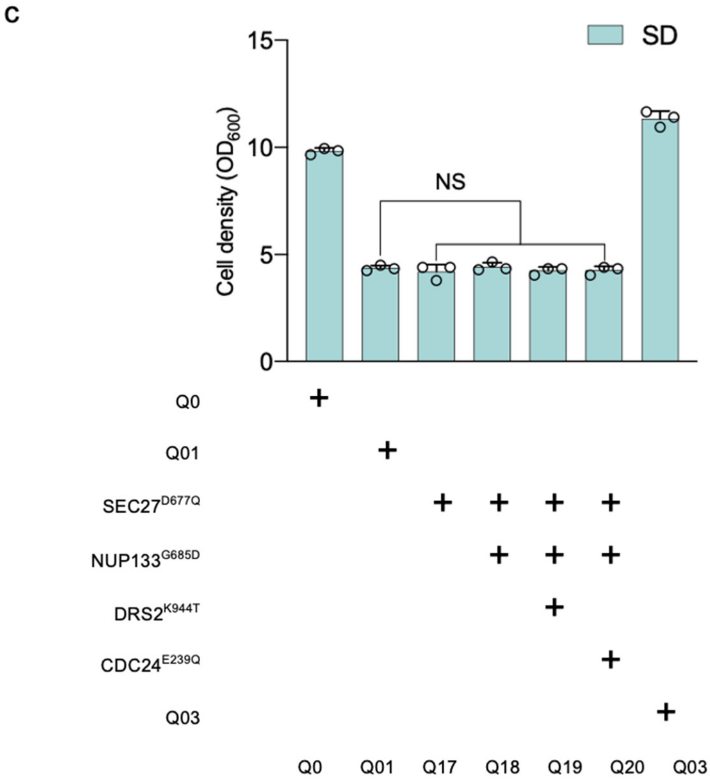

Fig. S4. Functional validation of adaptive mutations via reverse metabolic engineering.

a. Diagnostic PCR verification of evolved strains. **b**. Summary of missense mutations identified in candidate genes across evolved strains. **c**. Growth comparison of reconstructed mutants in SD medium at 30 °C for 96 h; Q0 as control. **d**. Growth stability of evolved strain Q03 over 7 additional passages, cell growth in SD medium (20g/L glucose) at 30 °C for 96 h.

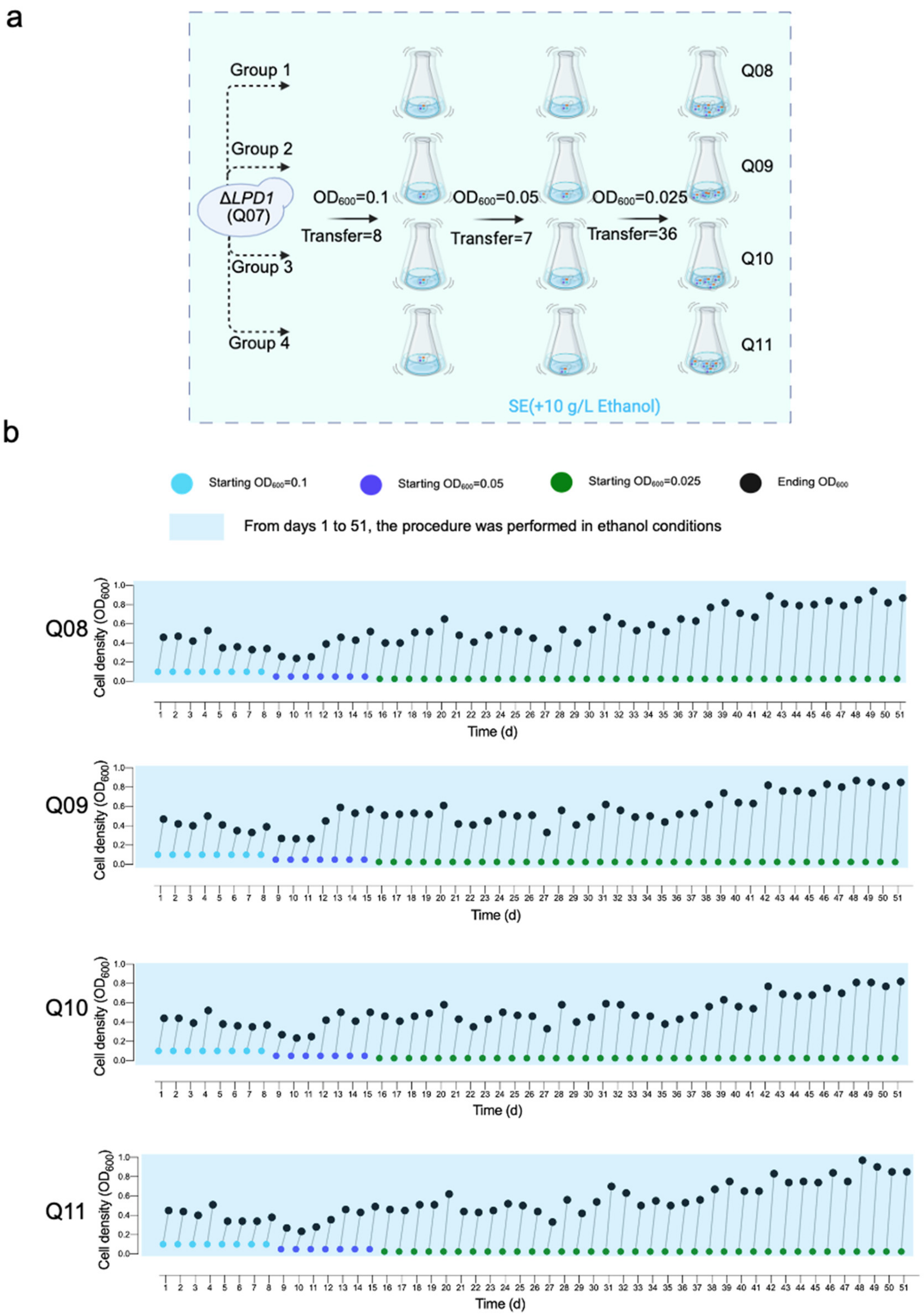

Fig. S5. ALE of the *Δlpd1* strain.

a. Schematic of the ALE procedure. **b**. Growth curves of *Δlpd1* strains during ALE. four independent colonies of *Δlpd1* strains were cultivated in SE with ethanol. Cultures were transferred daily to fresh medium.

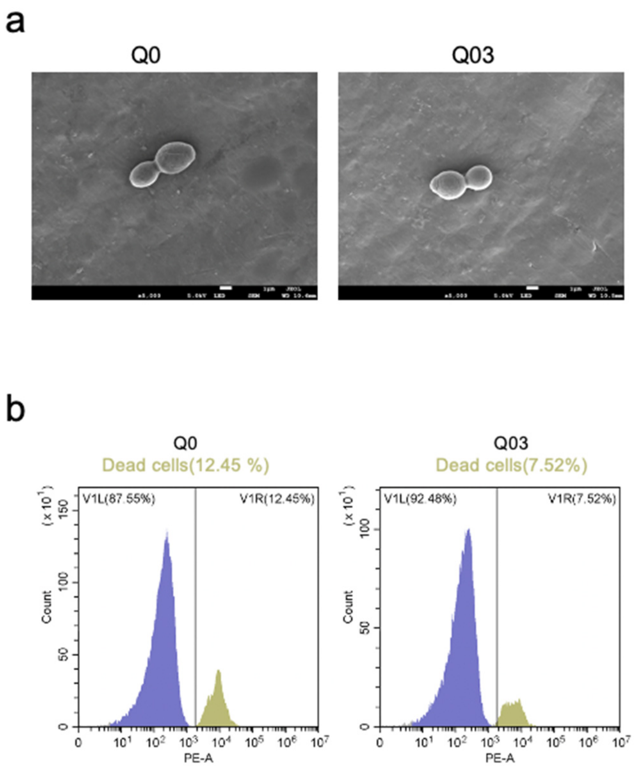

Fig. S6. Morphological and viability analysis of evolved and parental strains.

a. Scanning electron microscopy images showing the cell morphology of control (Q0) and evolved (Q03) strains. **b**. Flow cytometry plots showing proportions of dead cells in Q0 and Q03 strains after 96 h of cultivation in SD medium (20g/L glucose) at 30 °C. Dead cells were stained with PI and gated based on fluorescence intensity.

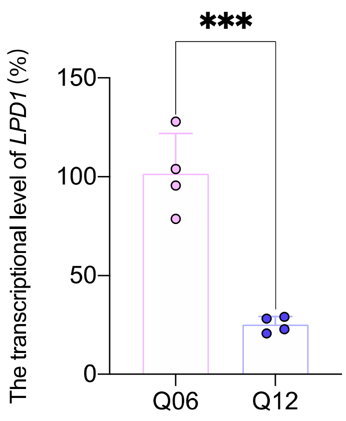

Fig. S7. The transcriptional level of *LPD1* in Q12, with the starting strain Q6 as the control.

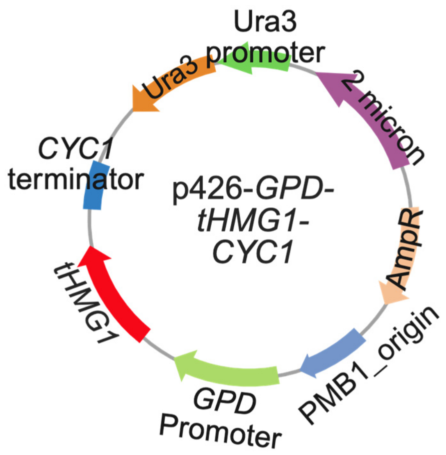

Fig. S8. Construction of the plasmid for tHMG1 overexpression. Schematic representation of the p426-GPD-tHMG1 plasmid used for overexpressing the truncated HMG1 gene (*tHMG1*) in *S. cerevisiae.*

Table S1. Strains used in this study.

Strain No.	Genotype / containing plasmid	Reference
CEN.PK 530-1D	(*MATa SUC2 MAL8C ura3-52 tpi1*(*41−707*):*loxP-KanMX4-loxP*)	[1]
CEN.PK 113-5D	*MATa SUC2 MAL8C ura3-52*	Lab stock
L01	Q0; *Δlpd1*	This study
L02	Q0; *Δsmx2*	This study
Q0	CEN.PK 530-1D; pCP-Aamylase	[2]
Q01	Q0; ΔSUT526	[2]
Q02	Q01, evolved	This study
Q03	Q01, evolved	This study
Q04	Q01, evolved	This study
Q05	Q01, evolved	This study
Q06	Renamed from CEN.PK 113-5D	Lab stock
Q07	Q06; *Δlpd1*	This study
Q08	Q07, evolved	This study
Q09	Q07, evolved	This study
Q10	Q07, evolved	This study
Q11	Q07, evolved	This study
Q12	Q03; *loxP-KanMX4-loxP::TPI1*; eliminated pCP-Aamylase	This study
Q13	Q06; p426-*GPD*p-*tHMG1-CYC1*t	This study
Q14	Q12; p426-*GPD*p-*tHMG1-CYC1*t	This study
Q15	Q13;*ADH1*p*::TEF1*p	This study
Q16	Q14;*ADH1*p*::TEF1*p	This study

Q17	Q01; SEC27^{D677Q}	This study
Q18	Q17; NUP133^{G685D}	This study
Q19	Q18; DRS2^{K944T}	This study
Q20	Q18; CDC24^{E239Q}	This study

Table S2. Plasmids used in this study.

**Plasmid**	**Relevant characteristics/ genotype**	**Reference**
pCP-Aamylase	CPOTud-(*TPI1*p-*alpha factor leader-amylase gene-TPI1*t)	[3]
p426-GPD	2μm, AmpR, *URA3*, *GPD*p, *CYC1*t	[4]
p426-GPD-tHMG1	2μm, AmpR, *URA3*, *GPD*p, *CYC1*t, *GPD*p-*tHMG1-CYC1*t	This study
pCas9	2μm ampR *TEF1*p-*Cas9 SNR52*p	[5]
pScURA	PCR template for gRNA-URA3-*SNR52*p-tGly	[5]
pYZ463	2μ,AmpR,URA3,*TEF1*p-*Cas9-CYC1*t, and *SNR52*p-*Not1-SUP4*t	[6]
pYZ463-ADH2	2μ,AmpR,URA3,*TEF1*p-*Cas9-CYC1*t, and *SNR52*p-*ADH2-SUP4*t	This study
pCas9-LPD1	2μm ampR *TEF1*p-*Cas9 SNR52*p-*LPD1*-gRNA	This study
pCas9-SMX2	2μm ampR *TEF1*p-*Cas9 SNR52*p-*SMX2*-gRNA	This study
pYZ463-SEC27	2μ,AmpR,URA3,*TEF1*p-*Cas9-CYC1*t, and *SNR52*p-*SEC27-SUP4*t	This study
pYZ463-NUP133	2μ,AmpR,URA3,*TEF1*p-*Cas9-CYC1*t, and *SNR52*p-*NUP133-SUP4*t	This study
pYZ463-DRS2	2μ,AmpR,URA3,*TEF1*p-*Cas9-CYC1*t, and *SNR52*p-*DRS2-SUP4*t	This study
pYZ463-CDC24	2μ,AmpR,URA3,*TEF1*p-*Cas9-CYC1*t, and *SNR52*p-*CDC24-SUP4*t	This study

Table S3. Primers used in this study.

Primer	Squence(5'-3')
tHMG1-OE-F	tggatcccccgggctgcaggaattcATGgaccaattggtgaagactg
tHMG1-OE-R	cgaggtcgacggtatcgataagcttttaggatttaatgcaggtgacgg
sgt2F-LPD1	AAAGGTCTCAGATCGCTGTTAAGCAATTAACTGGGTTTTAGAGCTAGAAATAGCAAGT
sgt2F-SMX2	AAAGGTCTCAGATCCAACTCCATAATATCCCTAGGTTTTAGAGCTAGAAATAGCAAGT
sgt2R	AAAGGTCTCA TGCGCAAGCCCGGAATCGAACCGGG
sgt3F	AAAGGTCTCACGCAGTTTTAGAGCTAGAAATAGCAAGTTAAAATAAGGC
sgt3R	AAAGGTCTCAAAACCTAGACACAGGGTAATAACTGATATAATTAAATTGAAGCTC
LPD1-P-F	GCGGAGCAACTTAATGGA
LPD1-P-R	CTCAATACTAAGCGGTTCTTC
SMX2-P-F	CGGCATAGGACAGGATTAC
SMX2-P-R	GGCTTTGACTACGGTTGT
ΔLPD1-D-F	TTGTTCGATTGTCTCTGTCGTACCATCAAAGAACATACTAACAGTTCACAAAACAGGAA
ΔLPD1-D-R	AAGCGGTTCTTCATAAATATATATACTATACTGTTTATTATTTCCTGTTTTGTGAACTG
ΔSMX2-D-F	ATAGAGTATCCGGCATAGGACAGGATTACGCAAACACACGCACAGATACAAATTATATA
ΔSMX2-D-R	TATTGCTGTATAGTACATAGAGGTCGACATATATATATATATATATAATTTGTATCTGT
ADH2(P)-K-F	GATCAGTCTCGTGAAGTGGAgttttagagctagaaatagcaagttaaaa
ADH2(P)-K-R	TCCACTTCACGAGACTGATCgatcatttatctttcactgcggagaagttt
ADH2(P)-D-F	CGGTTACAGCCTGTGTAACTGATTAATCCTGCCTTTCTAATgcacacaccatagcttcaaa
ADH2(P)-D-R	TCGTAGAAGATAATGGCTTTTTGAGTTTCTGGAATAGACATttgtaattaaaacttagatt
TEF1-P-F	ttcctctagggtgtcgtta
TEF1-P-R	ATCGCCGTACCACTTCAA

Supplementary References

1. Hou J, Tyo K, Liu Z, Petranovic D, Nielsen J: **Engineering of vesicle trafficking improves heterologous protein secretion in *Saccharomyces cerevisiae*.** *Metab Eng* 2012, **14:**120-127.
2. Qin L, Pan Y, Xue S, Yan Z, Xiao C, Liu X, Yuan D, Hou J, Huang M: **Multi-omics analysis reveals impacts of lincRNA deletion on yeast protein synthesis.** *Adv Sci* 2025**:**2406873.
3. Liu Z, Tyo KE, Martinez JL, Petranovic D, Nielsen J: **Different expression systems for production of recombinant proteins in *Saccharomyces cerevisiae*.** *Biotechnol Bioeng* 2012, **109:**1259-1268.
4. Dominik Mumberg RM, Martin Funk: **Yeast vectors for the controlled expression of heterologous proteins in different genetic backgrounds.** *Gene* 1995, **156:**119-122.
5. Zhang Y, Wang J, Wang Z, Zhang Y, Shi S, Nielsen J, Liu Z: **A gRNA-tRNA array for CRISPR-Cas9 based rapid multiplexed genome editing in Saccharomyces cerevisiae.** *Nat Commun* 2019, **10:**1053.
6. Zhao Y, Coelho C, Lauer S, Majewski M, Laurent JM, Brosh R, Boeke Jef D: **CREEPY: CRISPR-mediated editing of synthetic episomes in yeast.** *Nucleic Acids Research* 2023, **51:**e72-e72.
